# Job loss from poor health, smoking and obesity: a national prospective survey in France

**DOI:** 10.1136/jech.2007.060772

**Published:** 2008-03-13

**Authors:** F Jusot, M Khlat, T Rochereau, C Serme

**Affiliations:** 1 Institut de Recherche et Documentation en Economie de la Santé (IRDES), Paris, France; 2 Institut National d’Etudes Démographiques (INED), Paris, France

## Abstract

**Background and objectives:**

Health selection into unemployment may be either direct or operate by reference to health-related behaviours rather than health per se (indirect selection). Panel data are desirable to investigate selection effects, and the two types of selection processes may be concurrent. We examine jointly the roles of health and health-related behaviours as precursors of unemployment, in order to disentangle direct from indirect selection processes.

**Design:**

The data of a multi-round nationally representative health survey in France were analysed longitudinally, based on three data collection rounds: 1992–5, 1996–8 and 2000–2. Following employees salaried in the private sector and aged 30–54 years at baseline, we explored through logistic regression the influence of non-optimal self-rated health, smoking and obesity on the risk of being found unemployed 4 years later.

**Results:**

After adjustment for self-rated health, obesity was found to be a significant precursor of unemployment in women, and heavy smoking had that role in men. After adjustment for smoking and obesity, poor health at baseline was found to be a significant precursor of unemployment in both genders.

**Conclusion:**

Those findings confirm the intrinsic role of poor health and of health-related behaviours as precursors of unemployment, with gender-specific patterns for the latter. Public policy prescriptions regarding employees’ protection from job insecurities should integrate appropriate accommodations of health limitations, and the personal factors underlying unfavourable work and health behaviours should be investigated, in order to thwart indirect selection phenomena.

Unemployment and various types of flexible employment are associated with bad health outcomes,^1^ ^2^ and this is often interpreted within the “social causation hypothesis.” From there, the idea that job insecurities should be a point of concern for public health policies is gaining ground in the epidemiological literature.^3^ And yet, in order to elaborate well founded policy prescriptions, the role of the second facet of the “causation-selection framework”—that is, the selection hypothesis, should be given more consideration.

On one hand, the direct selection hypothesis assumes that health exerts a direct influence on employment, with people coming to be unemployed or remaining unemployed because of a disease.^4^ Indeed, people who develop chronic illnesses during their working life may become less able to cope with the demands of the job, and this may be compounded by the time-consuming use of medical services and the frequent sick leaves. They are therefore more likely to lose their job or leave it, in relation to their health limitations. Even if they lose their job for other reasons, they may have more difficulties than the others in regaining employment.^5^

On the other hand, the idea that selection might operate by reference to health behaviours rather than health itself directs attention to another explanatory mechanism, which is that of indirect selection.^6^ The indirect selection process assumes that certain personality characteristics may influence both “employability” through work behaviour and “health” through health behaviour, therefore generating a fallacious association between unfavourable health behaviours and unemployment.^7^ It has also been argued that “the true causal relationship is that by which the personality trait affects both health and unemployment separately,”^7^ although those traits are “rarely spelt out in exact terms.”^4^ Therefore, one way to investigate indirect selection is to consider that health behaviours can be taken as proxy of the unobservable personal characteristics upon which they depend.

The scanty longitudinal studies on health selection have provided interesting evidence. In The Netherlands,^8^ Norway,^9^ ^10^ Finland,^11^ Sweden,^12^ Canada,^13^ the United States,^14^ Germany^15^ and some regions of France,^16^ mobility out of employment was found to be health selective. However, most of those studies have investigated transitions out of employment (that is, into either unemployment or inactivity), and only a few^11^^–^13 15 have specifically targeted the group of people losing their job from poor health, and willing to work again. In a few other prospective studies, an association has been reported between smoking and subsequent unemployment,^17^^–^20 and between obesity and unemployment.^21^ The interpretation of those findings is not straightforward, as: (1) the health factors underlying the transition into economic inactivity are likely to be more severe and perhaps different from those attached to mobility into unemployment, and therefore the picture is likely to be somewhat mixed, and (2) behavioural risk factors and health are likely to be strongly correlated, and therefore the direct role of health on transitions in and out of employment is difficult to disentangle from the indirect role of behavioural risk factors, as each may confound the other.

The aim of this study is to analyse jointly the roles of health, smoking and obesity as precursors of unemployment in France, in order to extricate the contribution of direct health selection to the association between unemployment and ill health from that of indirect selection. For this purpose, we build on the “National Health, Health Care and Insurance Survey,” which is the only follow-up health survey available at the national level, and on self-rated health as an indicator of global health, and investigate the transitions based on survey data collected during the 1990s for employees salaried in the private sector and aged 30–54 years at baseline.

## METHODS

### Data collection

The National Health, Health Care and Insurance Survey (ESPS: “Enquête sur la Santé et la Protection Sociale”) is a multi-round health interview survey of all residents in households with at least one member included in the EPAS (“échantillons permanents d’assurés sociaux”), which is an ongoing random sample of beneficiaries of the national health insurance funds. This survey covers more than 95% of French households, and is coordinated by the Institute for Research and Information in Health Economics (IRDES) in Paris.^22^

The ESPS was designed as a “snapshot” survey of the population health and healthcare consumption at regular intervals, and included every year one-fourth of the EPAS between 1992 and 1997, and one-half every two years between 1998 and 2002. At each survey round, about 40% of the households in the EPAS files could not be reached, owing partly to inevitable losses caused by incomplete data and wrong or outdated addresses. Seventy per cent of the households that were reached agreed to participate in the survey, and the participation rate increased over time.^22^

Between data collection rounds, a majority of the subjects remained in the EPAS sample, while new subjects were included, and others were removed. Given this scheme, respondents be followed up every four years, with a maximum of three participations between 1992 and 2002. On average, the follow-up rate between consecutive survey rounds was close to 55%, and we found that educational level had no influence on the likelihood of follow-up; neither had any of the health variables, except smoking, with heavy smokers being less likely to be followed up.^23^

We are focusing on the transitions from employment at time t to unemployment at time t+4 years, in relation with a set of independent variables observed at time t. In the survey questionnaire, the response options for occupational status were active, unemployed and inactive (including retired, students, military contingent, housewives and recipients of disability pensions). People who had declared they were active had to specify whether they were self-employed, salaried in the private sector or salaried in the public sector. Self-employed (independent professionals, farm managers, craftsmen and independent shop or business owners) were excluded from the study: indeed, they are neither subjected to the risk of being fired, nor entitled to unemployment benefits when they cease their professional activity. In the latter case, they are expected to declare that they are “inactive” rather than “unemployed.” In France, employees salaried in the public sector have permanents jobs and are therefore protected from unemployment. Below age 30, individuals who lose their job may resume training or formal studies, thereby exiting the labour force.^24^ At the opposite age scale, starting from age 55, individuals who lose their job may be exempted from job seeking while retaining unemployment benefits until they reach pension age and claim their pension entitlement.^25^ Therefore, we have selected the individuals meeting the following criteria: (1) they had to be salaried in the private sector, aged 30–54 years and with either an indefinite-term or a fixed-term contract at the starting point, (2) to be either still salaried or unemployed 4 years later, and (3) to have no missing value for health and behavioural risk factors at baseline.

Salaried employees who met the first condition but were found to be inactive or retired at the second point were excluded. Individuals may meet the inclusion criteria either for only one transition between two consecutive periods, or for both. In the latter case, one transition out of the two was randomly selected in order to avoid both the issue of autocorrelation of residuals and the bias in favour of the individuals who had remained employed. The study sample consisted of 2420 transitions for women and 3287 for men.

### Health measures

Self-rated health was assessed at the first point of each study period, by asking the respondents to rate their own health on a scale ranging from 0 to 10, and dichotomising the grades into greater or equal to 8 (“optimal health”) as opposed to lower than 8 (“non-optimal health”). In the 2002 survey round, both this question and the classic one with five response options (very good, good, average, bad, very bad) were asked, and the correspondence between the scores of “8 or above” and the responses “good” or “very good” was quite good.^23^

Alcohol consumption was not recorded in the survey. Smoking consumption was categorised into non-smoker, smoker of 20 cigarettes per day (a pack) or less (light or moderate smoker), smoker of more than 20 cigarettes per day (heavy smoker), ex-smoker. Body mass index, calculated from reported height and weight, was categorised as: less than 25 (underweight or normal), greater or equal to 25 and lower than 30 (overweight), above or equal to 30 (obese).

### Statistical methods of analysis

Logistic regression was firstly used to analyse the relation of behavioural risk factors (smoking, body mass index) to self-rated health at the same time (cross-sectional analysis), controlling for age (categorised into 5-year intervals) and education level (primary school; lower secondary; higher secondary; university). The next stage consisted of an analysis of the influence of behavioural risk factors and self-rated health assessed at time t on employment status at time t+4 years (longitudinal analysis). The control variables in the latter analysis were the following sociodemographic characteristics assessed at time t: age (categorised into 5-year intervals), type of job contract (fixed-term; indefinite-term), educational level (primary school; lower secondary; higher secondary; university), type of household composition (couple with children; lone person; lone mother or father with children; couple without children; other), and presence of children aged less than 6 years in the household. This approach led to the analysis of (1) the association between self-rated health at time t and employment status at time t+4 years, adjusted for smoking habits, obesity and sociodemographic variables (at time t); (2) the association between smoking habits at time t and employment status at time t+4 years, adjusted by self-rated health, obesity and sociodemographic variables (at time t); and (3) the association between obesity at time t and employment status at time t+4 years, adjusted by self-rated health, smoking habits and sociodemographic variables (at time t). All the analyses were made separately for men and women.

## RESULTS

Self-ratings of health were less favourable among women than among men, with no difference with respect to obesity, and a substantially higher proportion of heavy smokers among men than among women. More women than men were found to be unemployed 4 years after initial observation (table 1). The proportion of unemployed at time t+4 years was higher among women and men reporting non-optimal self-rated health and among obese women (table 2).

**Table 1:** Description of the study sample

	Women		Men	
	%	No	%	No
**Employment status at time t+4**				
Still employed	92.2	2231	95	3124
Unemployed	7.8	189	5.0	163
**Characteristics at time t**				
Self-rated health				
Score ⩾8 (optimal)	74.4	1800	80.0	2628
Score <8 (non-optimal)	25.6	620	20.0	659
Obesity				
Underweight or normal	74.6	1806	55.6	1826
Overweight	18.7	453	37.1	1218
Obese	6.7	161	7.4	243
Smoking				
Non-smoker	56.2	1360	32.6	1072
Former smoker	19.6	475	28.0	921
Smokes up to 1 pack/day	22.6	548	32.2	1059
Heavy smoker	1.5	37	7.2	235
All sample	100	2420	100	3287

Salaried employees in the private sector aged 30–54 years in Enquête sur la Santé et la Protection Sociale survey, 1992–5, 1996–8 and 2000–2.

**Table 2:** Proportion of unemployed at time t+4 years among salaried employees in the private sector aged 30–54 years at time t in ESPS* survey, 1992–5, 1996–8 and 2000–2

Characteristics at time t	Women		Men	
	%	95% CI	%	95% CI
**Self-rated health**				
Score ⩾8 (optimal)	6.3	5.2 to 7.5	4.3	3.6 to 5.1
Score <8 (non-optimal)	12.1	9.5 to 14.7	7.4	5.4 to 9.4
**Obesity**				
Underweight or normal	6.8	5.7 to 8.0	5.3	4.2 to 6.3
Overweight	9.3	6.6 to 11.9	4.8	3.6 to 6.1
Obese	14.9	9.4 to 20.4	3.3	1.1 to 5.6
**Smoking habits**				
Non-smoker	8.5	7.0 to 9.9	3.7	2.6 to 4.9
Former smoker	6.1	4.0 to 8.3	5.4	4.0 to 6.9
Smokes up to 1 pack/day	7.3	5.1 to 9.5	5.1	3.8 to 6.4
Heavy smoker	13.5	2.5 to 24.5	8.1	4.6 to 11.6
All sample	7.8	6.7 to 8.9	5.0	4.2 to 5.7

*ESPS, Enquête sur la Santé et la Protection Sociale.

The first regression analysis focused on the relation of behavioural risk factors (body weight and smoking) to self-rated health at time t (cross-sectional analysis). Both attributes turned out to be strongly related to health outcomes (table 3). Obese women were much more likely to report non-optimal health than those in the normal range, and men too, but to a lesser extent. Regular smoking was associated with a definite health disadvantage, but in men only, and that disadvantage extended to all types of smokers: former smokers, smokers of up to one pack per day and heavy smokers.

**Table 3:** Odds ratios associated with non-optimal self-rated health for behavioural risk factors at baseline time t (cross-sectional analysis)

Independent variables	Women	Men
	OR* (95% CI)	OR* (95% CI)
**Obesity**		
Underweight or normal	1.0	1.0
Overweight	1.2 (0.9 to 1.5)	1.0 (0.8 to 1.2)
Obese	2.7 (1.9 to 3.8)	1.6 (1.2 to 2.2)
**Smoking habits**		
Never-smoker	1.0	1.0
Former smoker	1.0 (0.8 to 1.3)	1.3 (1.0 to 1.6)
Smokes up to 1 pack/day	0.9 (0.7 to 1.2)	1.4 (1.1 to 1.8)
Heavy smoker	1.2 (0.6 to 2.5)	2.2 (1.6 to 3.1)

Private sector employees aged 30–54 years in Enquête sur la Santé et la Protection Sociale survey, 1992–5, 1996–8 and 2000–2.

*Adjusted for age and educational level.

The second regression analysis (table 4) focused on the relation of non-optimal health, regular smoking and obesity of employed individuals at baseline time t to unemployment observed at time t+4 years (longitudinal analysis), based on a regression model including both the sociodemographic factors and the health-related factors. After adjustment for self-rated health and smoking habits, obesity was a significant precursor of unemployment for women only. Men who were heavy smokers at time t were much more likely to have moved into unemployment at time t+4 years than the non-smokers, after adjustment for self-rated health and obesity. Lastly, a non-optimal self-rated health (graded as less than 8) was associated with significantly elevated odds ratios in women and in men, after adjustment for smoking habits and obesity.

**Table 4:** Odds ratios associated with being unemployed at time t+4 years for self-rated health and behavioural risk factors at baseline time t (longitudinal analysis)

Independent variables	Women	Men
	OR* (95% CI)	OR* (95% CI)
**Self-reported health**		
Score ⩾8	1.0	1.0
Score <8	1.7 (1.2 to 2.3)	1.5 (1.1 to 2.2)
**Obesity**		
Underweight or normal	1.0	1.0
Overweight	1.3 (0.9 to 1.9)	0.8 (0.6 to 1.2)
Obese	2.0 (1.2 to 3.4)	0.5 (0.2 to 1.0)
**Smoking habits**		
Non-smoker	1.0	1.0
Former smoker	0.8 (0.5 to 1.2)	1.4 (0.9 to 2.1)
Smokes up to 1 pack/day	0.9 (0.6 to 1.4)	1.3 (0.8 to 2.0)
Heavy smoker	1.7 (0.6 to 4.8)	1.8 (1.0 to 3.3)

Private sector employees aged 30–54 years in Enquête sur la Santé et la Protection Sociale survey, 1992–5, 1996–8 and 2000–2.

*Adjusted for age, educational level, type of job contract, household composition and presence of children of less than 6 years in the household.

## DISCUSSION

In France, the literature on unemployment and health is very limited,^26^ ^27^ and the most striking feature to date is the considerable mortality disadvantage of the unemployed, in comparison with other countries.^2^ ^28^ ^29^ In order to shed some light on the factors underlying this disadvantage, this study focused on health-related attributes and poor health, and their specific role as precursors of mobility from employment to unemployment four years later. Obese men and women and male heavy smokers were found to be much more likely to report non-optimal health in the cross-sectional analyses. Analysing the data longitudinally, we find that non-optimal health, obesity in women and heavy smoking in men are independently associated with subsequent unemployment.

One asset of the current study is its prospective nature: the health and behavioural risk factors of the individuals were collected while they were employed, and related to their employment status four years later. Some of the study limitations may have influenced our findings. One issue is the lack of information on the events that have occurred during the 4-year time period between the two survey rounds. This type of incompleteness of the biographical information is expected to confuse the picture and therefore to lead to an underestimation of the associations between labour market trajectories and health. The same reasoning applies to the lack of information on the circumstances of job loss (resignation, dismissal or layoff). Given the design of ESPS as a “snapshot” survey of the population health and healthcare consumption, no special efforts were made to track the respondents from one round to the other, and this explains the relatively low follow-up rate (about 55%). One concern is the potential attrition bias, even though our investigation of the underlying factors did not support the existence of differential attrition according to health. In fact, there were many reasons behind losses to follow-up (unavailability or absence of respondent at time of visit, respondent move, entire household move, outdated information in the administrative files, refusals), and those reasons were not necessarily related to health. However, heavy smokers were found to be more likely to be lost, and so were unemployed individuals. We may therefore anticipate an under-representation of heavy smokers having lost their job in the sample, leading inevitably to a weakening of the strength of the association between smoking and subsequent unemployment. Also, the lack of information on alcohol consumption is regrettable, as the level of consumption by men is particularly high in France in comparison with other European countries.^30^ Given that in France alcohol consumption is more frequent among smokers,^31^ a confusion between the role of tobacco and that of alcohol is not unlikely.

As expected, we did find an association between smoking habits and self-rated health on one hand, and obesity and self-rated health, on the other hand, and this accords with the literature.^32^ We next analysed jointly the survey information on self-rated health, smoking habits and obesity in order to gain additional insights into the health-related processes of job loss. By including the three factors in the same analysis, we were able to eliminate possible confounding, and extricate the specific influence of each of them on the transition to unemployment. Traditionally, the role of ill health is considered to be a reflection of direct selection, and smoking or obesity as the reflection of indirect selection. The mechanisms generally hypothesised for health selection are a reduction of productivity in relation to either ill health or certain health behaviours. West has, however, viewed health selection as a “profoundly social process of discrimination by health.”^6^ In fact, the word discrimination comes from the Latin “discriminare,” which means to “distinguish between,” and discrimination is conceptualised as an action based on prejudice resulting in unfair treatment of people. Distinctions between people that are based just on individual merit (such as personal achievement, skill or ability) are generally not considered socially discriminatory. Discrimination involves treating someone less favourably because of the possession of a prohibited (undesirable) attribute (among which disease or disability), regardless of his/her productivity. West suggests a number of possible dimensions of discrimination, and among them physical attractiveness.^6^ In our society, attractiveness for women is closely linked to slimness, and therefore obese women may suffer discrimination in the labour market because they do not live up to the gendered beauty norms for women in society.^19^ Discrimination with regard to smoking habits is less likely, although it may be hypothesised in some contexts.

Concerning tobacco, we did find that employed men who reported that they smoked heavily were much more at risk of being found to be unemployed 4 years later. In order to consider residual confounding from severe tobacco-related diseases which may have occurred between the survey rounds, we have re-run the analyses after including self-rated health at time t+4 years among the covariables. The findings remained essentially the same, which provides support for the hypothesis of an intrinsic role of tobacco consumption. A statistical association between smoking and subsequent unemployment has been reported in quite a few prospective studies.^17^^–^20 In France, one study has provided evidence in favour of a negative association between smoking and upward occupational mobility in the Gazel cohort of male employees of the national electricity and gas company.^33^ In the United States, Ryan *et al*^17^ observed that cigarette smoking at the time of hire was associated with elevated rates of accidents, absence, discipline, and sacking among postal workers, and hypothesise that smoking could indicate risk taking and susceptibility to accidents. This is somewhat consistent with the conclusion by Waldron and Lye,^34^ that “certain personal characteristics or early experiences influenced both smoking adoption and adult unemployment.” Lastly, in a study of youth unemployment in France,^24^ the author conclude that there is a subpopulation that is excluded from the market of long-term, steady jobs, and he relates this situation to personal characteristics determining some kind of intrinsic “employability.” Another health-related variable that stands out in our study is obesity, which is increasingly recognised as a public health problem in Europe.^35^ The prevalence of this condition is known to vary greatly according to social and economic factors, but in gender-specific ways. In women, studies in the developed world have consistently reported a powerful inverse relation between socioeconomic position and employment status and obesity: the higher the social status, the rarer obesity. For men, the association is less consistent, as it can be positive, negative or absent.^35^^–^37 We found that obese women faced an odds ratio for mobility out of employment of about 2, which is considerable. Obesity though may be related to a previous history of unemployment, as certain subpopulations experience long-term exclusion from the labour market.^24^ In the literature, longitudinal evidence on obesity and employment is scarce. The only piece of evidence available in France is based on retrospective information from the 2003 Decennial Health Survey, in which obese men and women had a much higher percentage of working years spent unemployed and a much lower probability of regaining employment than the non-obese men and women.^21^ The authors conclude in favour of a significant stigmatisation, and even discrimination against obese people, to be attributed to the relative rarity of this condition in France, especially in comparison with the United States. In a cohort study from Sweden, being overweight at age 16 and at age 21 was related to future working-class position among women only, which is interpreted as gendered discrimination, “based on the societal norms for female bodies in our societies.”^19^

Concerning the role of ill health, we found that non-optimal self-rated health was a significant precursor of unemployment for men and women, with odds ratios around 1.5, after adjustment for smoking and obesity. Those findings accord with prospective studies of health and subsequent unemployment or unstable employment in Finland,^11^ Canada^13^ and Sweden,^12^ and we further demonstrate that the role of health is intrinsic and not confounded by indirect selection processes operating through health behaviours. A priori, women may be more vulnerable than men in the presence of ill health, as they have to exercise multiple roles.^38^ In France particularly, women have a higher participation in the labour market than in other European countries.^39^ We did not find any significant difference in the amount of health selection among women compared to men, and neither did van de Mheen *et al* in The Netherlands,^8^ considering the role of less-than-good self-perceived health in association with subsequent unemployment or inactivity. In Germany, Arrow reported that health selection only concerned women and foreign men, considered as “vulnerable” groups.^15^ The findings for men and women may be difficult to compare; in our survey, about one-quarter of the women reported non-optimal health, as opposed to one-fifth of the men, and this may reflect different perception of health and diseases.^40^ In addition, women are subjected in France to other factors of disadvantage in the labour market, as they consistently have higher unemployment rates than men, and are much more frequently involved in part-time jobs,^39^ which may be viewed as an adaptation to the multiple role constraints.

Public policy recommendations concerned with workers’ protection from job insecurities should include proper consideration of the disadvantage in the labour market of those with worse health and health risks. The handling of direct selection issues involves the implementation of properly targeted measures to accommodate the health limitations of workers and improve their “employability.” As far as indirect selection is concerned, more research is urgently needed to gain insight into the common denominators of health and work behaviours, and in the long run develop appropriate programmes to help individuals towards a better adjustment in the labour market and a safeguarding of their health capital. Discrimination phenomena against obese women or individuals suffering from health problems should also be more thoroughly investigated. Detailed analyses of health trajectories and the development of behavioural risk factors in relation to employment trajectories would provide useful insight into the complexities of individuals’ experiences.

**What is already known on this subject**

- Some studies have demonstrated that employees reporting poor health are more at risk of job loss, and this is interpreted as “direct” health selection.
- Similarly, smokers and obese workers were found to face the same type of risk, and this is interpreted as “indirect” health selection.
- Possible confounding between the effects of health and those of health behaviours has not been properly considered in those studies.

**What this study adds**

- Using prospective data from France, we conducted a joint analysis of the influence of health, smoking habits and obesity of employees at a given time on the probability of being unemployed 4 years later, in order to separate direct from indirect selection.
- We found concurrent evidence for direct and indirect selection processes: (1) non-optimal self-rated health is associated with job loss in men and women; (2) there are gender-specific associations for health-related behaviours, with a significant role of obesity in women and of heavy smoking in men.

**Policy implications**

- The findings concerning direct selection support the implementation of properly targeted measures to accommodate the health limitations of workers and improve their “employability.”
- The findings concerning indirect selection support the development of research into the personal traits underlying behaviours related to both the health and work spheres, and into discrimination processes operating in the labour market.
